# Supplementary material for: Removing unwanted variation with CytofRUV to integrate multiple CyTOF datasets
Source: eLife. 2020 Sep 7;9:e59630. doi: 10.7554/eLife.59630 (PMC7500954; doi:10.7554/eLife.59630)
Supplement: Transparent reporting form [file elife-59630-transrepform.docx]

***eLife’s* transparent reporting form**

We encourage authors to provide detailed information *within their submission* to facilitate the interpretation and replication of experiments. Authors can upload supporting documentation to indicate the use of appropriate reporting guidelines for health-related research (see [EQUATOR Network](http://www.equator-network.org/%20)), life science research (see the [BioSharing Information Resource](https://biosharing.org/)), or the [ARRIVE guidelines](http://www.plosbiology.org/article/info:doi/10.1371/journal.pbio.1000412) for reporting work involving animal research. Where applicable, authors should refer to any relevant reporting standards documents in this form.

If you have any questions, please consult our Journal Policies and/or contact us: [editorial@elifesciences.org](mailto:editorial@elifesciences.org).

**Sample-size estimation**

- You should state whether an appropriate sample size was computed when the study was being designed
- You should state the statistical method of sample size computation and any required assumptions
- If no explicit power analysis was used, you should describe how you decided what sample (replicate) size (number) to use

Please outline where this information can be found within the submission (e.g., sections or figure legends), or explain why this information doesn’t apply to your submission:

No sample size estimation was necessary as the purpose of our study is to exhibit some of the batch effects that can arise with CyTOF experiments using three different datasets.

**Replicates**

- You should report how often each experiment was performed
- You should include a definition of biological versus technical replication
- The data obtained should be provided and sufficient information should be provided to indicate the number of independent biological and/or technical replicates
- If you encountered any outliers, you should describe how these were handled
- Criteria for exclusion/inclusion of data should be clearly stated
- High-throughput sequence data should be uploaded before submission, with a private link for reviewers provided (these are available from both GEO and ArrayExpress)

Please outline where this information can be found within the submission (e.g., sections or figure legends), or explain why this information doesn’t apply to your submission:

- We described in the “Results” section the number of batches of each dataset we have analyzed (First dataset: lines 123-126; Second dataset: lines 363-365; Third dataset: lines 392-398).
- We have described all the information and details of biological and technical replicates in our dataset in the Flow Repository database under accession number FR-FCM-Z2L2. The reviewers have full access to all of the generated data at the <http://flowrepository.org/id/RvFrVB2mAm2q08TjrWBEaJ4scGfMTPDwZiToOS22zcMztKarmVH563xYyFW78ZAL>. We also described it in the “Methods” section (lines 614- 639 and lines 648). In addition, we also included the information related to the two other datasets taken from those used in (Schuyler et al., 2019) and in CytoNorm (Van Gassen et al., 2019) in the “Results” section (Second dataset: lines 363-365; Third dataset: lines 392-398).
- We described in the “Results” section the number of samples, replicates and batches of each dataset we have analyzed (First dataset: lines 123-126; Second dataset: lines 363-365; Third dataset: lines 392-398).
- Handling of outliers: Not applicable.
- Datasets were included as described on pages 4,5, 11 and 12 of the ms. No data were excluded.
- Our dataset is available in the Flow Repository database and therefore the reviewers have full access to all of the generated data, under accession number FR-FCM-Z2L2 at the <http://flowrepository.org/id/RvFrVB2mAm2q08TjrWBEaJ4scGfMTPDwZiToOS22zcMztKarmVH563xYyFW78ZAL>.

**Statistical reporting**

- Statistical analysis methods should be described and justified
- Raw data should be presented in figures whenever informative to do so (typically when N per group is less than 10)
- For each experiment, you should identify the statistical tests used, exact values of N, definitions of center, methods of multiple test correction, and dispersion and precision measures (e.g., mean, median, SD, SEM, confidence intervals; and, for the major substantive results, a measure of effect size (e.g., Pearson's r, Cohen's d)
- Report exact p-values wherever possible alongside the summary statistics and 95% confidence intervals. These should be reported for all key questions and not only when the p-value is less than 0.05.

Please outline where this information can be found within the submission (e.g., sections or figure legends), or explain why this information doesn’t apply to your submission:

- All the statistical analysis methods and computation of metrics are described in the Methods section of the manuscript (line 525-596). The pipeline and scripts used to generate the results described in this manuscript is available in the supplementary data.
- Data before CytofRUV normalisation is presented in Figure 1, Figure 2, Figure 3A, 3B, 3C, Figure 4, Figure 6A, 6B, Supp. Fig.1a, Supp. Fig. 2, Supp. Fig. 3A, Supp. Fig.4, Supp. Fig. 9A.
- The statistical test used for differential analysis is described in the “Methods” section (lines 586-596).
- We did not compute p-values in our study.

(For large datasets, or papers with a very large number of statistical tests, you may upload a single table file with tests, Ns, etc., with reference to sections in the manuscript.)

**Group allocation**

- Indicate how samples were allocated into experimental groups (in the case of clinical studies, please specify allocation to treatment method); if randomization was used, please also state if restricted randomization was applied
- Indicate if masking was used during group allocation, data collection and/or data analysis

Please outline where this information can be found within the submission (e.g., sections or figure legends), or explain why this information doesn’t apply to your submission:

- We described in the “Results” section the sample type of each sample in each dataset (First dataset: lines 123-126; Second dataset: lines 363-365; Third dataset: lines 392-398) and also in the Table 1.
- No masking took place.

**Additional data files (“source data”)**

- We encourage you to upload relevant additional data files, such as numerical data that are represented as a graph in a figure, or as a summary table
- Where provided, these should be in the most useful format, and they can be uploaded as “Source data” files linked to a main figure or table
- Include model definition files including the full list of parameters used
- Include code used for data analysis (e.g., R, MatLab)
- Avoid stating that data files are “available upon request”

Please indicate the figures or tables for which source data files have been provided:

- We did not upload the source data files but we provided the pipeline and scripts used to generate the results and figures described in this manuscript in the supplementary data folder.
- Details about the pipeline and R scripts used to generate the results and figure described in this manuscript are available in the README.txt file in the supplementary data folder.
- The package is available at: www.github.com/mtrussart/CytofRUV. Installation and R code usage instructions for both the R package and the R-Shiny application can be found on the GitHub page.
